# A total infectome approach to understand the etiology of infectious disease in pigs

**DOI:** 10.1186/s40168-022-01265-4

**Published:** 2022-05-10

**Authors:** Xinyi Huang, Weichen Wu, Xiaoxiao Tian, Xin Hou, Xingyang Cui, Yihong Xiao, Qiulin Jiao, Pei Zhou, Liqiang Liu, Weilin Shi, Ligong Chen, Yue Sun, Yongbo Yang, Jianxin Chen, Guihong Zhang, Jinling Liu, Edward C. Holmes, Xuehui Cai, Tongqing An, Mang Shi

**Affiliations:** 1 State Key Laboratory of Veterinary Biotechnology, Harbin Veterinary Research Institute, Chinese Academy of Agricultural Sciences, Harbin, China; 2 School of Medicine, Shenzhen campus of Sun Yat-sen University, Sun Yat-sen University, Shenzhen, China; 3 College of Animal Science and Veterinary Medicine, Shandong Agricultural University, Tai’an, China; 4 College of Veterinary Medicine, South China Agricultural University, Guangzhou, China; 5 College of Life Sciences and Food Engineering, Hebei University of Engineering, Handan, China; 6 Harbin Weike Biotechnology Development Company, Harbin Veterinary Research Institute, Chinese Academy of Agricultural Sciences, Harbin, China; 7 College of Veterinary Medicine, Hebei Agricultural University, Baoding, China; 8 College of Animal Science and Veterinary Medicine, Shenyang Agricultural University, Shenyang, China; 9 Sydney Institute for Infectious Diseases, School of Life & Environmental Sciences and School of Medical Sciences, The University of Sydney, Sydney, Australia

**Keywords:** Infectome, Meta-transcriptomics, Animal disease, Pathogens

## Abstract

**Background:**

The global pork industry is continuously affected by infectious diseases that can result in large-scale mortality, trade restrictions, and major reductions in production. Nevertheless, the cause of many infectious diseases in pigs remains unclear, largely because commonly used diagnostic tools fail to capture the full diversity of potential pathogens and because pathogen co-infection is common.

**Results:**

We used a meta-transcriptomic approach to systematically characterize the pathogens in 136 clinical cases representing different disease syndromes in pigs, as well as in 12 non-diseased controls. This enabled us to simultaneously determine the diversity, abundance, genomic information, and detailed epidemiological history of a wide range of potential pathogens. We identified 34 species of RNA viruses, nine species of DNA viruses, seven species of bacteria, and three species of fungi, including two novel divergent members of the genus *Pneumocystis*. While most of these pathogens were only apparent in diseased animals or were at higher abundance in diseased animals than in healthy animals, others were present in healthy controls, suggesting opportunistic infections. Importantly, most of the cases examined here were characterized by co-infection with more than two species of viral, bacterial, or fungal pathogens, some with highly correlated occurrence and abundance levels. Examination of clinical signs and necropsy results in the context of relevant pathogens revealed that a multiple-pathogen model was better associated with the data than a single-pathogen model was.

**Conclusions:**

Our data demonstrate that most of the pig diseases examined were better explained by the presence of multiple rather than single pathogens and that infection with one pathogen can facilitate infection or increase the prevalence/abundance of another. Consequently, it is generally preferable to consider the cause of a disease based on a panel of co-infecting pathogens rather than on individual infectious agents.

**Video abstract**

**Supplementary Information:**

The online version contains supplementary material available at 10.1186/s40168-022-01265-4.

## Background

Pigs are economically important domestic animals that account for over 30% of meat production worldwide [1]. Despite their importance, the productivity of the global swine industry is continually affected by infectious diseases. Large-scale recurring epidemics caused by pathogens such as African swine fever virus (ASFV) [2], porcine reproductive and respiratory syndrome virus (PRRSV) [3, 4], porcine epidemic diarrhea virus (PEDV) [5, 6], and classical swine fever virus (CSFV) [7, 8] have resulted in massive mortality, trade restrictions, and major reductions in production rate [9]. A wide variety of low virulent pathogens, such as pseudorabies virus (PRV) [10] and porcine parvovirus (PPV) [11], are also present in local swine herds and either cause mild disease manifestations that can result in embryonic death and affect pig growth and meat production or impact disease severity as a co-infecting or opportunistic pathogen, such as porcine circovirus 2 (PCV2) [12, 13].

The recent deployment of pathogen discovery technology has identified several epidemics or local outbreaks of emerging pathogens, such as Reston ebolavirus [14] and swine acute diarrhea syndrome coronavirus [15, 16]. These pathogens most likely originated in one mammalian species (such as bats) and were introduced into pigs via cross-species transmission; thus, they also have the potential to infect humans [17]. To date, more than 50 recognized viral pathogens and 46 bacterial pathogens have been associated with pig diseases [18, 19]. However, while most have been described in independent research studies, little attention has been paid to investigating the simultaneous presence and dynamics of these pathogens in pig populations [13, 20–22].

Under field conditions, infectious diseases in pigs are often characterized by multiple occurrences of different pathogens within the same individual (i.e., co-infection) and do not easily fit a “one disease, one pathogen” paradigm [13, 23]. Co-infection is common in swine production systems under intensive and packed production conditions [24]. Viruses such as PRRSV, PCV2, and swine influenza A virus (swine IAV) and bacteria such as *Streptococcus suis* and *Mycoplasma hyopneumoniae* are often simultaneously detected in pigs with respiratory symptoms [13, 25–28] and can result in severe disease [29–38]. In one study of piglets experiencing diarrhea, an average of five and a maximum of ten virus species were identified [39]. Although it is unclear whether all pathogens present in a sample contribute to disease manifestation, co-infecting pathogens may act synergistically, leading to either disease aggravation [26, 33, 40] or chronic forms of disease [41, 42].

Despite numerous efforts to investigate the diversity and complexity of pathogens in pigs, few studies have attempted a comprehensive characterization of all pathogens and their interactions in pigs [13]. Herein, we used a meta-transcriptomics sequencing approach to reveal all viral, bacterial, and fungal pathogens within individual pigs (i.e., their total infectome) in a systematic manner so that they can be characterized simultaneously in the context of specific disease syndromes. Specifically, we investigated 136 clinical cases of different diseases in pigs and revealed the diversity, abundance, and genomic information of each pathogen type. Using these data, we also investigated their interactions, potential clinical manifestations, and epidemiological impact.

## Results

### The “total infectome” of diseased pigs

Between March 2018 and July 2019, we investigated 136 cases of swine disease (i.e., the “diseased group”) experienced on pig farms from 15 major swine production provinces across China (Additional file 1). Disease severity varied from mild herd illness and growth retardation to large-scale animal mortality. Disease-relevant tissue samples from these outbreaks, including lung, intestine, lymph nodes, liver, spleen, kidney, and mixed tissues, were collected from deceased animals. For comparison, corresponding tissue samples were collected from 12 non-diseased animals (i.e., the “healthy group”), including six from facilities that raise specific pathogen free (SPF)-grade animals for research purposes. Subsequently, we performed total infectome analysis using an unbiased meta-transcriptomics (i.e., total RNA sequencing) approach to reveal all the viruses, bacteria, and eukaryotic pathogens present in each individual sample.

**Fig. 1:**
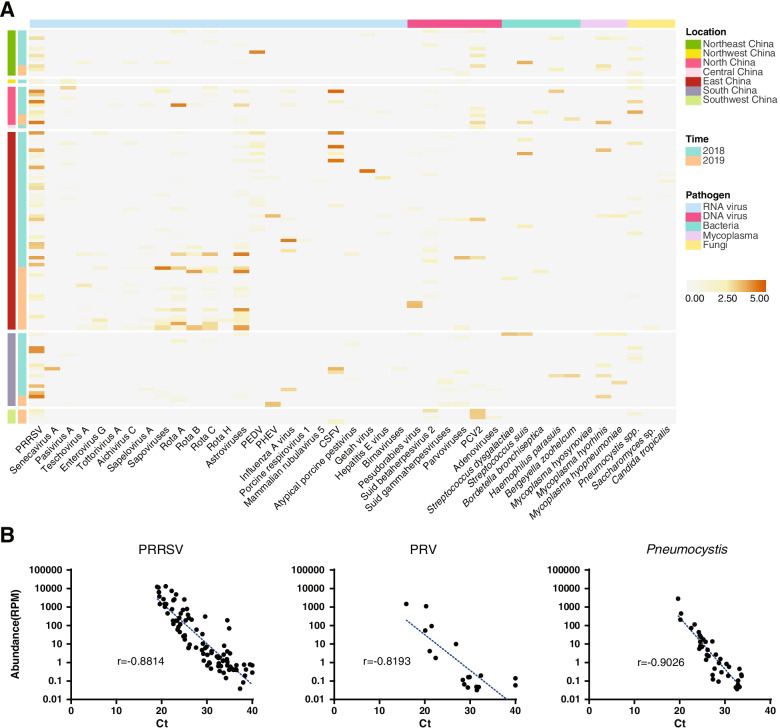
Total infectome characterization in 136 clinically diseased pigs. **A** Heat map showing the prevalence and abundance of pathogens in each of the cases. The pathogens (*x*-axis) are divided into five different groups—RNA viruses, DNA viruses, bacteria, mycoplasma, and fungi. The cases (*y*-axis) are divided based on sampling year and locations, including seven regions across China. **B** Comparisons of pathogen abundance measured by qPCR and meta-transcriptomics approaches. Abundance by RT-qPCR methods (*x*-axis) is measured by cycle threshold, (i.e., Ct value), while those by meta-transcriptomics (*y*-axis) are measured by RPM in log10 scale. The comparisons are performed on three representative pathogens: PRRSV (RNA virus), PRV (DNA virus), and *Pneumocystis* (fungi)

Pathogens were identified by directly comparing sequencing reads against the non-redundant protein, bacterial genome, and universal Cox1 gene databases and confirmed by genome mapping and qPCR (or RT-qPCR) assays. Here, we only considered (i) known pathogens, (ii) opportunistic pathogens, or (iii) uncharacterized viruses related to pathogens that have the potential to cause diseases in mammals. Overall, 34 species of RNA viral pathogens, 9 species of DNA viral pathogens, 7 species of bacteria and mycoplasma pathogens, and 3 species of fungal pathogens were identified in 109 of the 136 total cases examined in this study (Fig. 1A, Additional file 1). Among these were microbes known to cause acute or severe diseases, such as PRRSV, CSFV, PEDV, *M*. *hyopneumoniae*, *Haemophilus parasuis*, and zoonotic pathogens such as *S*. *suis*, PRV, hepatitis E virus (HEV), rotavirus A, and Japanese encephalitis virus (JEV). Although the abundance level of JEV (0.19–0.48 reads per million, RPM) was below our threshold (i.e., 1 RPM) to be officially classified as positive, its presence was confirmed by RT-qPCR (Additional file 2). The vast majority of pathogens identified, such as sapoviruses (five species); astroviruses (six species); parvoviruses (four species); herpesviruses (three species); porcine respirovirus 1; rotaviruses B, C, and H; and *Mycoplasma hyorhinis*, were likely opportunistic or only associated with mild and subclinical disease in pigs.

**Fig. 2:**
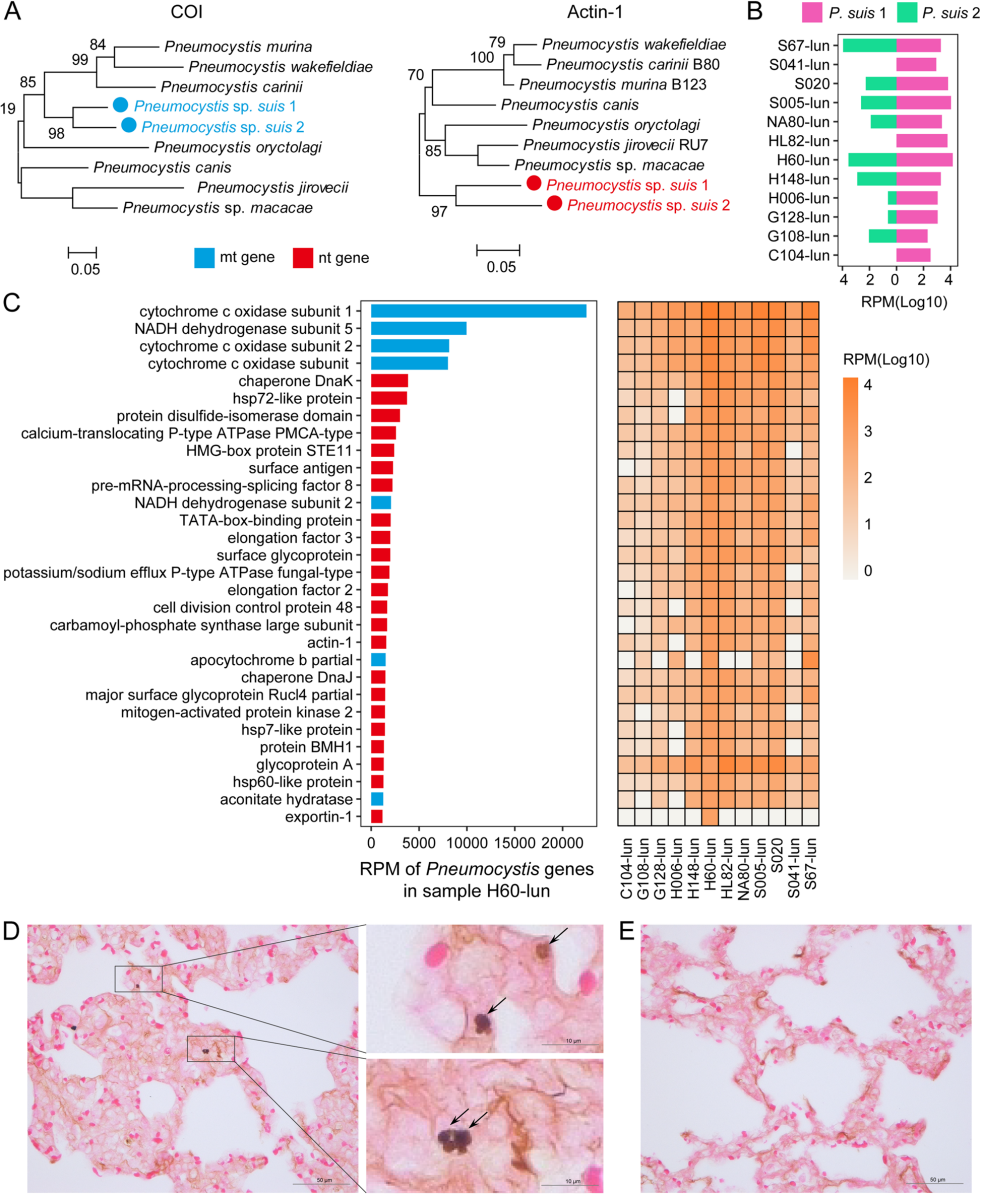
The diversity, prevalence and gene expression profiles of the two *Pneumocystis* spp. identified in this study. **A** Evolutionary history of the two *Pneumocystis* spp. within the context of existing members of genus *Pneumocystis*. The phylogenetic trees were estimated using a maximum likelihood method on the COI and Actin-1 genes. **B** Bar graph of the presence and abundance of each type of *Pneumocystis* identified here. **C** Gene expression profiles (measured in RPM) for *Pneumocystis* in sample H60-lun (left panel) and all the other samples (right panel). The presence of *Pneumocystis* was confirmed by GMS staining of **D***Pneumocystis* positive and€) *Pneumocystis* negative samples

In addition to the well-known swine pathogens described above, we discovered two divergent members of the fungal genus *Pneumocystis*, associated with pneumonia in mice and humans, with a high prevalence and abundance in the lungs of diseased pigs (Fig. 2). Analyses of the sample (H60-lun) with the highest *Pneumocystis* abundance revealed two separate lineages that shared 88.48% nucleotide identity with each other and 81.65% and 81.20% identity with their closest relative, *Pneumocystis* sp. *macacae*. This divergence suggests that these lineages represent two different species. Based on the widely accepted trinomial nomenclature, we tentatively named these pathogens as *Pneumocystis* sp. *suis* 1 and 2 [43]. Phylogenetic analyses revealed a generally consistent topology in the mitochondrial and nuclear genes, in which these two new species formed a monophyletic cluster distinct from that of the *Pneumocystis* found in other mammalian species (Fig. 2A), consistent with previous findings based on ribosomal RNA genes [44]. Interestingly, the two *Pneumocystis* species identified here often co-appeared in the samples we examined (9/12) and were mostly associated with lung infection (Fig. 2B), although their corresponding abundance levels sometimes differed. Further characterization of the transcriptomic profiles of these two *Pneumocystis* spp. revealed the expression of a functionally diverse range of genes associated with energy metabolism, replication, structure, and cell survival (Fig. 2C), confirming that these are metabolically active microbes that exist at high abundance within the diseased host. Finally, the presence of *Pneumocystis* was confirmed by Grocott’s methenamine silver (GMS) staining, which showed a clustering of dark brown, ovoid *Pneumocystis* cysts (Fig. 2D) in samples that tested positive for *Pneumocystis* spp. but not in negative samples (Fig. 2E).

### Evaluation of infection status by quantification of pathogen transcriptomes

Marked variation in pathogen abundance, measured in RPM mapped to pathogen genomes (viruses and bacteria) or mitochondrial genomes (fungi), was observed. While we set the lowest threshold for pathogen detection at 1 RPM, the highest abundance reached 28,519 RPM (or 2.85% of total non-ribosomal RNA) and was observed in a lung sample infected with CSFV (Additional file 1). The highest abundance levels (> 10^5^ RPM) were always associated with RNA virus infection, although relatively high abundance levels (> 10^3^ RPM) were also frequently observed for transcripts from DNA viruses, bacteria, and fungi (Additional file 1). For the newly identified fungal *Pneumocystis* spp*.*, the highest abundance level was 2828 based on mapping against mitochondrial transcripts. To validate the presence and abundance of pathogens, we performed RT-qPCR or qPCR assays for PRRSV (RNA virus), PRV (DNA virus), and *Pneumocystis* spp. (fungi). The correlation between the RPM value and the threshold cycle (Ct) value estimated by RT-qPCR or qPCR assay was high (Pearson’s *r* = − 0.82 to − 0.90, Fig. 1B), suggesting that meta-transcriptomics produced a reliable estimate of pathogen abundance.

### Comparisons of infectomes in diseased and healthy pigs

**Fig. 3:**
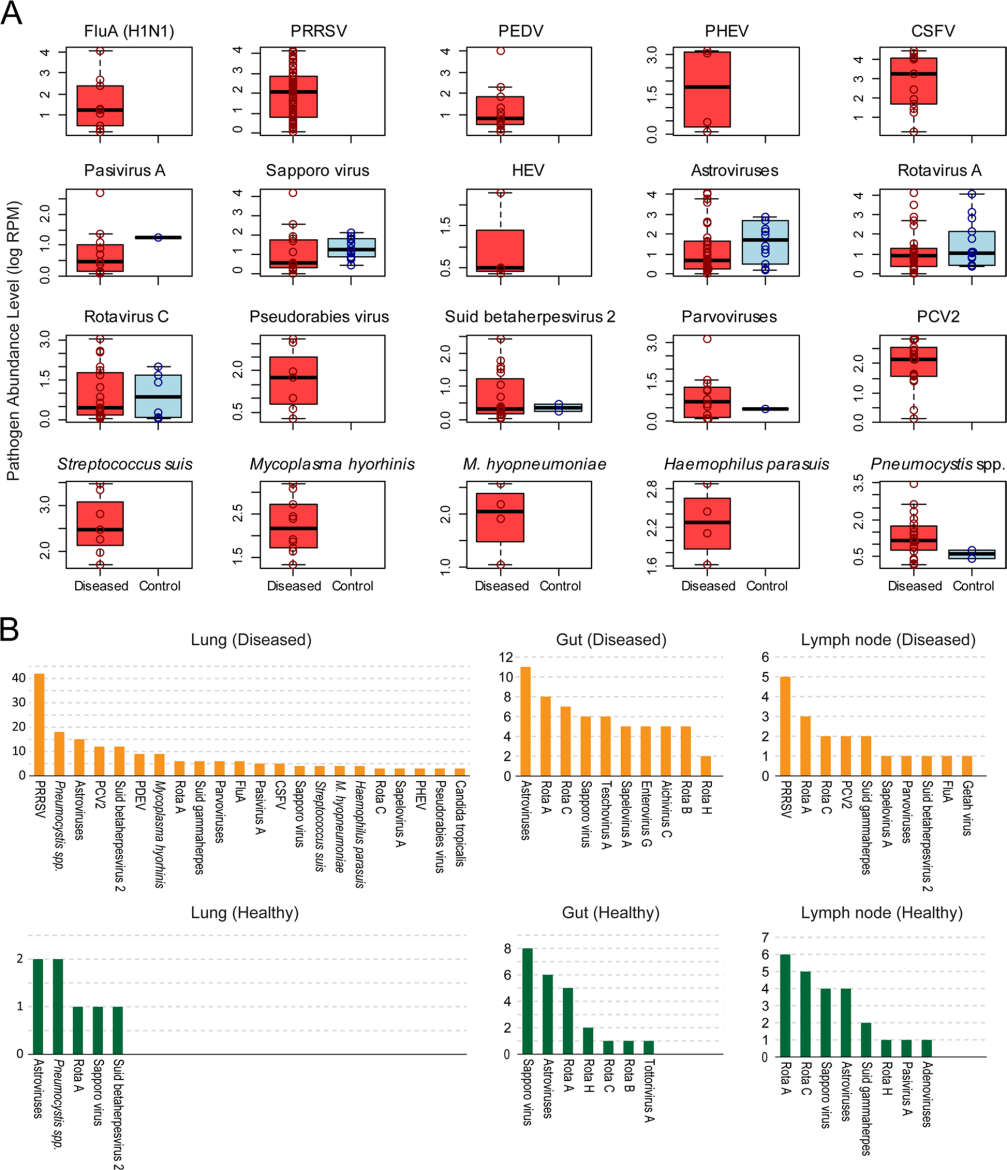
Disease association and tissue preference for the pathogens identified in this study. **A** Comparisons of pathogen abundance between the diseased and control groups. Each boxplot shows the abundance (measured by RPM) distribution of diseased (red) and control (blue) cases. **B** Bar graph showing the prevalence level of pathogens (y-axis) in different types of diseased and healthy tissues - lung, gut and lymph node

To establish the potential disease associations of the pathogens discovered in this study, we compared the prevalence and abundance of each pathogen in diseased and healthy pigs (Fig. 3A). In many cases, including swine IVA, PRRSV, PEDV, porcine hemagglutinating encephalomyelitis virus (PHEV), and PCV2, extremely low levels of abundance, or even complete absence, were observed in the healthy pig group, suggesting that these are *bona fide* pathogens. Conversely, a small number of viruses appeared in both the diseased and healthy groups: sapoviruses, astroviruses, rotavirus A, and rotavirus C showed similar prevalence and abundance between the two groups, whereas Suid betaherpesvirus 2, parvoviruses, and the newly identified *Pneumocystis* spp. had much lower prevalence and abundance in healthy pigs than in diseased pigs (Fig. 3A). We also identified the possible organ/tissue tropisms for each pathogen (Fig. 3B). For example, PRRSV and PCV2 were mainly detected in the lungs and lymph nodes, whereas pasivirus A and the newly identified *Pneumocystis* spp. were strictly limited to the lungs. Nevertheless, some pathogens commonly associated with enteric diseases, namely astroviruses, rotaviruses, and Sapporo virus, also appeared in other organs from both the healthy and diseased groups, although generally at low abundance (Fig. 3B).

### In-depth genomic analysis of swine diseases

**Fig. 4:**
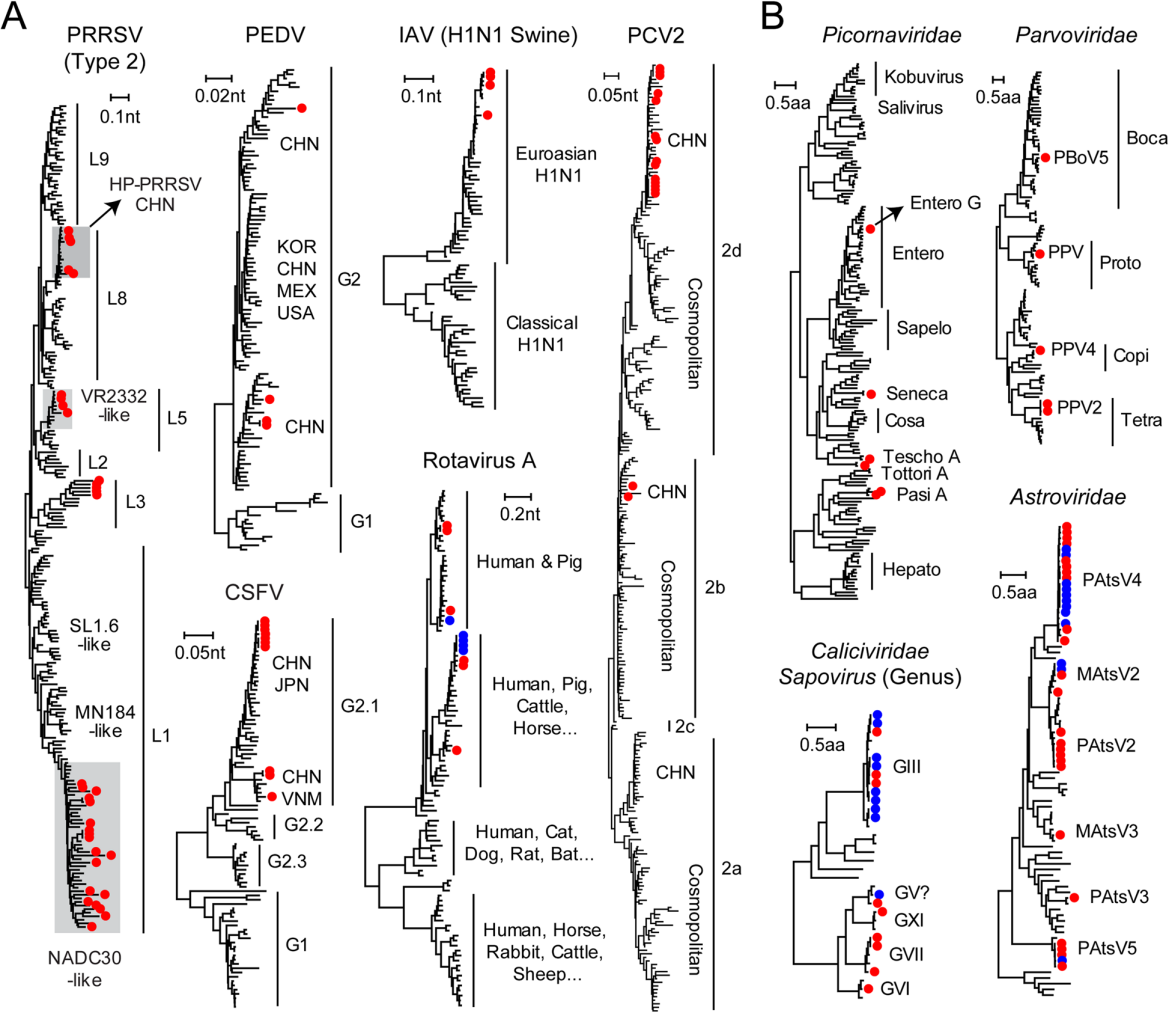
Phylogenetic diversity of the viruses identified in this study. Species level (**A**) and family level (**B**) maximum likelihood phylogenetic trees. Sequences identified from the diseased groups are marked with a red solid circle, whereas those from healthy controls are marked with a blue solid circle. For clarity, sequence names were not shown on the tree. The corresponding taxonomy/lineage and geographic information are provided on the right of the tree

Our meta-transcriptomics analyses enabled an in-depth characterization of all the pathogens present in the animals studied here, including detailed genotyping/subtyping, estimation of intra-specific diversity, and identification of temporal-geographic patterns (Fig. 4). Notably, multiple genotypes/subtypes with distinct pathogenicity and transmissibility were identified. For example, both type 1 and 2 PRRSV were detected (Fig. 4A, Additional file 3), and we identified four lineages (lineages 1, 3, 5, and 8) within the type 2 PRRSV (Fig. 4A). While lineage 5 is largely associated with the modified live vaccine Ingelvac PRRS® MLV, the other three lineages have either been endemic in China for over 20 years (lineages 3 and 8) or recently introduced to China (lineage 1) [45]. Among the PRRSV, higher pathogenicity was associated with lineages 1 and 8, often referred to as NADC30-like and HP-PRRSV, respectively, which comprised the majority of the cases (24 out of 33; Fig. 4A). Similarly, more than two genetic variants were identified for each of the pathogens known to be associated with acute or severe diseases, namely PEDV, CSFV, IVA, PCV2, and Getah virus, which were most closely related to viruses identified within China or from neighboring Asian countries (Fig. 4A, Additional file 3), indicative of regionalized transmission. Substantial diversity at both inter-specific and intra-specific levels was also observed in the case of the less pathogenic virus families/genera, such as those from *Astroviridae* and sapovirus (*Caliciviridae*) (Fig. 4B). Indeed, we identified more than six species of astrovirus and five species of sapovirus, many of which shared < 90% nucleotide similarity with existing sequences, meriting the designation of new genotypes or subtypes. Among these, porcine astrovirus 4 was more commonly detected than other astrovirus species and was shed by both healthy and diseased swine. Sapovirus genogroup III was the predominant variant detected in the present study. Finally, the viruses identified in healthy pigs were very closely related to those found in diseased animals in the case of rotaviruses, sapoviruses, and astroviruses (Fig. 4 and Additional file 3), again suggesting that these variants/species were unlikely to cause overt diseases on their own.

### Microbial co-infection and infectome complexity

**Fig. 5:**
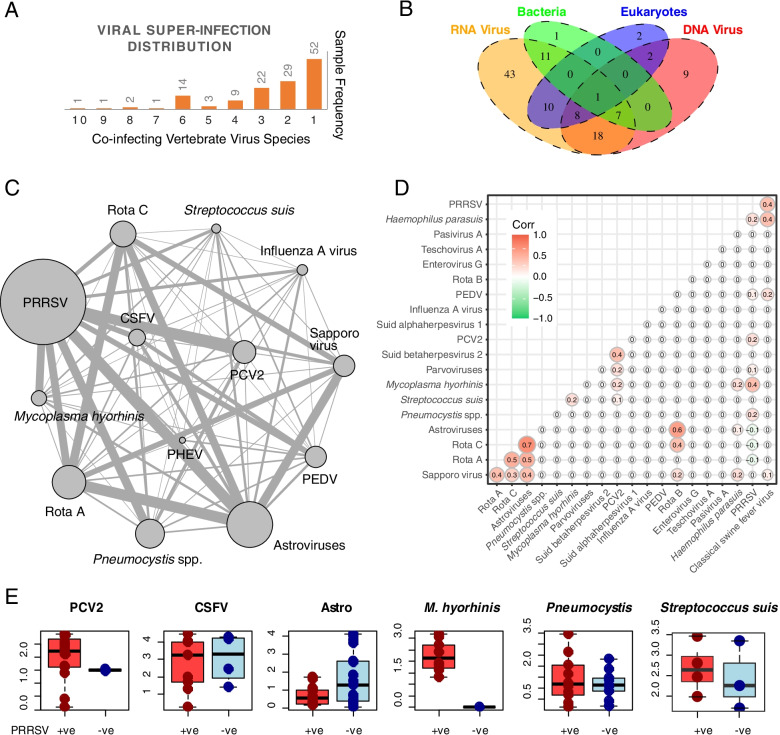
Co-infection characteristics and associations within the infectome. **A** Distribution of viral co-infections. **B** Co-infection frequencies among four different categories of pathogen—RNA virus, DNA virus, bacteria, and fungi. **C** Co-infection network based on frequencies estimated between each pair of important pathogens. **D** Correlation of abundance levels among different pathogens. **E** Boxplot showing the abundance distribution and prevalence (number of dots) comparisons of four viruses—PCV2, CSFV, astroviruses—*M*. *hyorhinis*, and *Pneumocystis* between PRRSV positive and PRRSV negative samples

Microbial co-infections were common in the diseased pigs examined in this study. In the case of viruses, 82 samples were infected with at least two virus species compared with 52 samples infected with only one virus (Fig. 5A). Interestingly, 22 samples were co-infected with more than five viruses. Adding to this complexity were co-infections among viruses, bacteria, and fungi (Fig. 5B), revealing highly complex infectomes for diseased pigs that may not be easily explained by a single pathogen–single disease model.

We further estimated the co-infection frequency of each pair of pathogens. This revealed higher frequencies of co-infection with PRRSV than of co-infection without PRRSV, although PRRSV was the most prevalent pathogen in our dataset (Fig. 5C). Indeed, for a number of respiratory pathogens, such as PCV2, *M*. *hyorhinis*, and *H*. *parasuis*, the majority of the cases had co-infection with PRRSV. Despite the dominance of PRRSV, viruses commonly associated with the digestive tract—rotaviruses, sapoviruses, and astroviruses—had a higher frequency of co-infection with each other than with PRRSV (Fig. 5C). Furthermore, positive correlations in the abundance (*p* < 0.05) of these viruses were found, indicating that their infection within the gut sometimes occurred synchronously. For example, in sample S144-gut from a pig experiencing acute digestive symptoms, we identified ten pathogens associated with the digestive tract, four of which were highly abundant: sapporovirus (330 RPM), rotavirus B (1,091 RPM), rotavirus C (354 RPM), and astroviruses (4,127 RPM) (Additional file 1). Relatively high positive correlations (*p* < 0.05) were also observed between PRRSV and *M*. *hyorhinis*, PRRSV and CSFV, and CSFV and *H*. *parasuis* (Fig. 5D). Nevertheless, the presence of PRRSV was associated with an increased prevalence and/or abundance of some other pathogens, such as PCV2, CSFV, and *M*. *hyorhinis*, although no effect was observed with pathogens such as astroviruses and *Pneumocystis* spp. (Fig. 5E)*.*

### Clinical manifestations

**Fig. 6:**
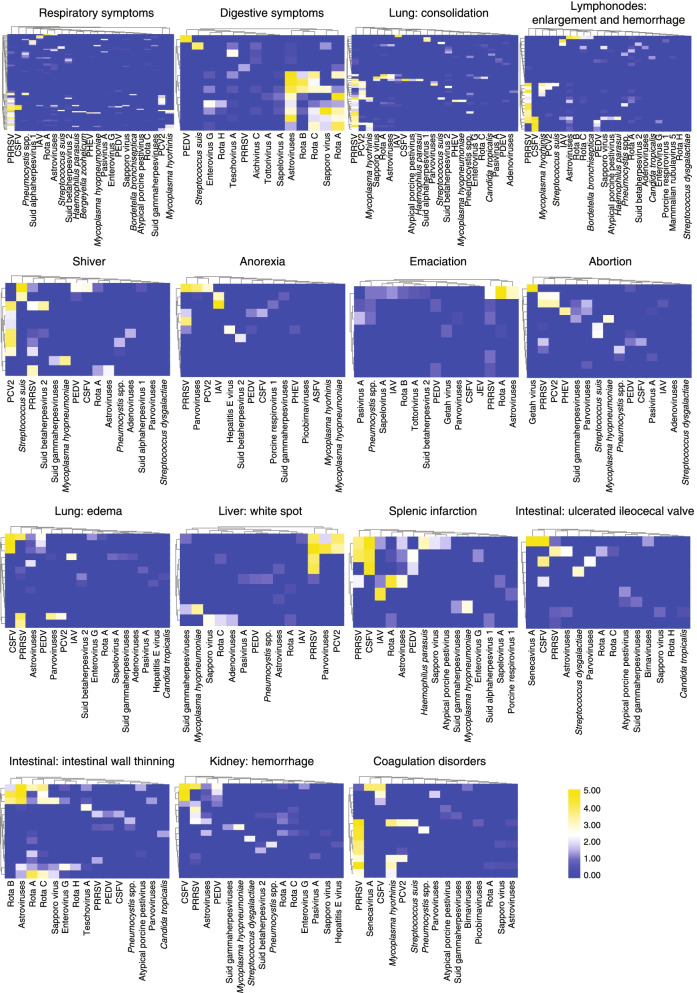
Pathogens associated with 15 disease types in pigs. For each of the disease types, a heat map displays the prevalence and abundance of the pathogens identified. Disease types include general symptoms (e.g., respiratory symptoms, digestive symptoms), specific symptoms (e.g., shiver, abortion, emaciation), as well as autopsy information (e.g., lung consolidation, kidney hemorrhage)

The complexity of infectomes often makes determining the causal relationship between the pathogen and disease challenging. Therefore, we examined the clinical symptoms and necropsy results in the context of a panel of relevant pathogens instead of individual microbes (Fig. 6). Compared with the single-pathogen model (Additional file 4), the infectome model provided more complete information on pathogen–disease relationships (Fig. 6). For respiratory symptoms, PRRSV and a number of co-infecting pathogens such as PCV2 and *M*. *hyorhinis* were the main contributors to the disease, although other pathogens such as *Pneumocystis* spp., pseudorabies virus, and swine H1N1 influenza A viruses were also frequently detected in the PRRSV-negative cases. Conversely, digestive symptoms or an “intestinal wall thinning” syndrome was often characterized by a combination of more than four digestive system-associated pathogens, many at high abundance (Fig. 6). Furthermore, 16 of the 25 cases with PRRSV and CSFV, singly or in combination, were associated with “lymph node enlargement and hemorrhage,” “splenic infarction,” “renal hemorrhage,” or “coagulation disorder.” Five of six cases infected with PCV2 (alone or with PRRSV) were associated with “shiver,” a neurological symptom. Finally, the “abortion” symptoms in our study were seemingly associated with a number of pathogens, among which PRRSV, PCV2, Getah virus, PHEV, and *S*. *suis* were present in relatively high abundance.

## Discussion

We have revealed total infectomes associated with diseased pigs in China, identifying complex interactions of multiple infectious agents within which co-infections are common [13, 23]. Although sequencing data alone is unlikely to reveal the clinical consequences and fine mechanisms for every pathogen combination, we examined potential models of pathogen interaction. First, we considered the single-pathogen model, which assumes that one major pathogen contributes to most disease manifestations and that the remaining pathogens having a negligible effect on the main clinical symptoms. This was the case when the pigs were infected with highly pathogenic viruses, such as ASFV, and we expected to observe the dominance of the causative agent over other pathogens. For example, case JL89, presenting with severe digestive symptoms, was infected with PEDV (9,230 RPM) alone. However, the single-pathogen model is relatively uncommon in our data, and most cases were characterized by the presence of other pathogens that are likely relevant to disease manifestation. For example, the case of S023, in which the animal experienced abortion, was characterized by a high frequency of Getah virus in the lungs (18,672 RPM); however, PEDV was also present (3 RPM), suggesting that its clinical symptoms might have reflected virus co-infection. Furthermore, even for highly virulent pathogens such as CSFV (12,267–28,517 RPM), disease-relevant co-pathogens such as PRRSV (2549–7392 RPM) and *H*. *parasuis* (737 RPM) often appeared within individual animals, again suggesting that the single-pathogen model is too simplistic.

As most cases (88/136) were associated with at least two relevant pathogens, our data favor a model in which the interaction among co-infecting pathogens may contribute to disease manifestation in pigs, although these pathogens can independently induce disease. Indeed, it has been demonstrated under both experimental and field conditions that infections with two or more disease-relevant pathogens, including virus–virus [13, 20, 21, 29, 33, 35, 37, 38, 41, 42, 46–50], virus–bacteria [13, 23, 31, 32, 34, 36, 51–60], and bacteria–bacteria [13, 30, 61–63] co-infections, can result in changes in pathogen replication, more severe clinical outcomes, or longer disease duration than that of individual infections alone. The mechanism of increased pathogenicity is complex and largely depends on the specific pathogens involved [13, 23]. Generally, an impaired host immune system, damaged epithelial barrier, and excessive inflammatory response can result in complex infection scenarios and worse clinical outcomes [23].

One of the most important co-infecting pathogens in pigs is PRRSV. This virus was not only ubiquitous in diseased pigs but also associated with an increase in the prevalence and/or abundance of other viral and bacterial pathogens, such as PCV2 and *M*. *hyorhinis* (Fig. 5E); in this study, *M*. *hyorhinis* was identified in ten pigs, all of which contained PRRSV as a co-pathogen. Indeed, co-infections involving PRRSV are well documented, and PRRSV, which targets macrophages and dendritic cells in the respiratory tract, is generally believed to be able to impair the host immune system, resulting in increased susceptibility to other pathogens [64–66].

Another interesting observation was that many pathogens associated with enteric diseases, particularly astroviruses, Sapporo virus, picornaviruses, and rotaviruses, also appeared in healthy controls, even in SPF pigs, at relatively high abundance levels (> 100 RPM). Since the enteric diseases described here as well as in previous studies were usually characterized by concurrent infections with multiple pathogens, these enteric pathogens may act in a synergistic or additive manner such that only multiple pathogens can lead to overt or severe enteric symptoms [67]. This is supported by the simultaneous occurrence and high abundance correlation of astroviruses, sapoviruses, picornaviruses, and rotaviruses in the diseased samples (Fig. 5). As these co-infecting pathogens usually show similar clinical signs, achieving an accurate diagnosis based on clinical presentation alone is often difficult.

We identified two divergent species in the genus *Pneumocystis* that may be potential respiratory fungal pathogens in pigs. *Pneumocystis* has been identified in a variety of mammalian species, including humans, and is usually an opportunistic infection of the lungs. *Pneumocystis* infections have been frequently detected in diseased and healthy pigs, and surveys conducted in Denmark, Japan, Korea, and Brazil revealed a 7–60% positive rate [44, 68–71]. While a few studies have suggested a lack of association with pulmonary lesions [44, 72], others have revealed an important link to disease outbreaks [69, 73]. Although *Pneumocystis* was present in both healthy and diseased samples of the pigs studied here, it was at a significantly elevated abundance in diseased animals. Furthermore, *Pneumocystis* was identified with other pathogens, including PRRSV (*n* = 10), pasivirus A (*n* = 5), and PCV2 (*n* = 4), suggesting that *Pneumocystis* infection may follow primary viral infections in these cases. While its disease manifestation requires further investigation, the *Pneumocystis* identified in pigs is highly divergent from *Pneumocystis carinii*, requiring a new species designation.

Notably, we also identified several zoonotic pathogens that pose a potential threat to human health. Among these, *S*. *suis*, which causes sporadic human outbreaks in Asian countries, with a mortality rate as high as 19.2% [74], was identified in 7/136 cases presented here and in multiple Chinese provinces. In addition, we detected a number of vector-borne viruses (i.e., JEV and Zika virus) for which pigs may act as intermediate or amplifying hosts. Interestingly, a latent virus present at high prevalence in the pig population, PRV, is now associated with human endophthalmitis [75]. In addition, we identified porcine respirovirus 1, which shared the highest relative (77.1% identity) to human parainfluenza virus 1 [76]. Since porcine organs are considered favorable resources for xenotransplantation [77], a particular concern is that viruses characterized by both ubiquitous presence and latent infection can result in disease manifestations after transplantation. Indeed, some opportunistic pathogens were present in SPF pigs. Hence, a thorough pathogen investigation is required to exclude any potential threats to organ recipients.

Our study highlights the power of meta-transcriptomics to understand the etiological basis of infectious diseases in pigs. Importantly, this approach can (i) reveal all types of pathogens in a single assay, showing that multiple infections are the norm rather than the exception; (ii) provide accurate estimations of pathogen abundance; and (iii) help identify each pathogen at the most precise taxonomic level using the genomic sequences obtained, which enables scrutiny of detailed epidemiological history. Collectively, the total infectome revealed by meta-transcriptomics represents a powerful model for understanding infectious diseases in domestic animals, which goes beyond pathogen discovery and characterization.

## Conclusions

Here, we describe the total infectome associated with diseased pigs in China, revealing a huge diversity of viruses, bacteria, and eukaryotic pathogens. Although most of these are well-known swine pathogens, we identified two divergent species in the genus *Pneumocystis* associated with respiratory symptom in pigs. More importantly, our findings revealed that most of the pig diseases examined were better explained by the presence of multiple rather than single pathogens and that infection with one pathogen can facilitate infection or increase the prevalence/abundance of another pathogen, resulting in more complicated and severe clinical manifestations in diseased pigs. Therefore, it is generally preferable to consider the cause of a disease based on a panel of co-infecting pathogens rather than individual infectious agents. In summary, our study highlights the complexity of the infectome in each pig disease syndrome and underlines the importance of performing comprehensive pathogen characterization before a diagnosis is made.

## Methods

### Sample collection

A total of 185 tissue samples from 136 diseased pigs were collected in China between March 2018 and July 2019 (Additional file 1). The sample collection covered 15 provinces, especially those with major swine industries, including Shandong, Guangdong, Hebei, and Heilongjiang. For comparison, six SPF-grade and six clinically healthy piglets were collected from Heilongjiang Province. The SPF piglets, which were provided by the National Science and Technology Infrastructure Centre (Harbin), were free of 23 main pathogens, which was confirmed using RT-qPCR and/or ELISA. These pathogens include ten viruses, namely foot-and-mouth disease virus (serotypes A and O), transmissible gastroenteritis virus, PCV2, PRRSV, PRV, CSFV, PEDV, JEV, swine IAV, and ASFV; ten bacteria, namely *Pasteurella multocida*, *Bordetella bronchiseptica*, *M*. *hyopneumoniae*, *Actinobacillus pleuropneumoniae*, *Brucella*, *Brachyspira hyodysenteriae*, *Salmonella*, *H*. *parasuis*, *S*. *suis*, and *Leptospira*; and three parasites, *Toxoplasma gondii*, lice, and *Sarcoptes scabiei*. Tissue samples from the lung, spleen, intestine, liver, kidney, and lymph nodes were collected from both diseased and healthy pigs. The tissues were first processed to remove irrelevant connective tissue and blood vessels. A small piece of tissue (< 0.5 cm) was subsequently cut from the bottom and immediately immersed in RNAlater™ (Invitrogen, Waltham, MA, USA) to prevent RNA degradation. All samples were transported on dry ice and stored at − 80 °C until further processing.

### Meta-transcriptomic sequencing

After thawing on ice, the tissues were rinsed with sterile phosphate-buffered saline (PBS) to remove blood and contaminants from the surface. Subsequently, a piece of tissue was cut from the bottom of the sample and homogenized in PBS. Total RNA was extracted from the homogenate using TRIzol (Invitrogen), followed by ribosomal RNA (rRNA) removal using a RiboZero Gold Kit (Human/Mouse/Rat) and Trueseq (Illumina, San Diego, CA, USA) RNA library construction. The libraries were then subjected to 150 bp pair-end sequencing on the Illumina Hiseq 4000 or NovaSeq platform (Illumina).

### Pathogen discovery and characterization

The sequencing results were subjected to quality control procedures, including the removal of low-quality reads, adaptor sequences, non-complex reads, and duplicated reads, using the BBmap software package (https://sourceforge.net/projects/bbmap/). Pig rRNA reads were subsequently removed by mapping against a comprehensive rRNA sequence collection downloaded from the SILVA database (http://www.arb-silva.de) [78]. The remaining reads were either (i) directly compared against the non-redundant protein (nr) database using Diamond Blastx [79], with an *e* value threshold set at 1E-5, or (ii) assembled into contigs using Megahit [80] before comparisons against the nr database.

For virus identification, taxonomic information was obtained for each of the blast hits, and those that matched the kingdom “Viruses” were retained. False-positives and endogenous virus elements were identified and subsequently removed by comparing the reads and contigs against the non-redundant nucleotide (nt), whole genome shotgun, and vector sequence databases. Virus contigs/genomes with < 90% amino acid similarity to known viruses were treated as potential novel virus species. For bacterial and fungal identification, we first used MetaPhlAn2 [81] to identify potential pathogens in both groups. Relevant background bacterial and fungal mitochondrial genomes were downloaded from NCBI/GenBank and used as templates for read mapping and abundance estimation. Based on the mapping results for each case, we generated relevant contigs (i.e., consensus sequences) for Blastn comparisons against the nt database to determine microbial taxonomy at the species level. Highly divergent fungal species were first identified based on Blastx analyses against the nr database and then confirmed by comparing key genes (e.g., *COI* and *Actin*-*1*) against sequences from related taxa.

The abundance level for each virus genome was calculated using the following formula: total viral reads/total non-redundant reads × 1,000,000 (i.e., RPM). The abundance levels of bacterial and fungal pathogens were calculated as RPM based on mapped reads against the relevant bacterial and fungal mitochondrial genomes, respectively. A pathogen was considered as “positive” in a sample if its abundance level was greater than 1 RPM. To identify potential false-positives resulting from index hopping, we used a threshold of 0.1% for pathogens present in the libraries from the same sequencing lane: any read numbers < 0.1% of the most abundant library were treated as “negative.”

### Virus genome confirmation using RT-qPCR and nested RT-PCR assays

Genomic sequences were confirmed by mapping reads against assembled contigs for each mammalian-associated virus identified in this study. RT-qPCR assay confirmation was performed on representative RNA viruses (i.e., PRRSV) [82] or qPCR for DNA viruses (PRV) [83] and fungi (*Pneumocystis*) [84] (Additional file 5). The same sample RNA used for meta-transcriptomic analysis was also subjected to primers designed for a specific or related group of pathogens. Accordingly, 1 μg of total RNA was used for reverse transcription (RT). First-strand cDNA was synthesized using M-MLV (TaKaRa, Dalian, China) according to the manufacturer’s instructions and then amplified using Premix Ex Taq (TaKaRa). A Ct value of less than 38 was considered positive. For pathogens with extremely low abundance levels (RPM < 1), namely JEV, a RT-qPCR assay was performed to confirm their presence within the sample [85].

### Evolutionary analyses

Pathogen genomes/genes were first aligned with related reference virus sequences downloaded from NCBI/GenBank using the progressive FFT-NS-i algorithm implemented in the MAFFT multiple sequence alignment program [86]. Ambiguously aligned regions were removed using the TrimAl program [87]. Maximum likelihood phylogenetic trees were estimated using PhyML [88], employing the GTR model of nucleotide substitution and SPR branch swapping in all cases. Support for individual nodes in the tree topology was estimated with an approximate likelihood ratio test using Shimodaira–Hasegawa-like procedures.

### Grocott’s methenamine silver staining

To confirm the presence of the newly discovered fungi *Pneumocystis* spp., qPCR-positive lung samples were fixed in 10% buffered formalin, embedded in paraffin, sectioned to 4 μm, and stained with a commercial GMS staining kit (Baso Biotech Ltd, Zhuhai, China) according to the manufacturer’s instructions.

### Data availability

All sequencing reads were deposited in the SRA database under the project accession number PRJNA800593. Relevant pathogen genome/gene sequences have been deposited in GenBank under accession numbers OM201171-OM201230, OM151337, and OM149829-OM149830.

## Supplementary Information

**Additional file 1.** Table showing sample collection, pathogen abundance, and clinical signs.

**Additional file 2.** RT-qPCR result showing the presence of JEV in sample NM84. Top panel: amplification plot and standard curve based on absolute standards as well as in sample NM84 (green curve and blue square). Bottom panel: Melt curve plots for the sample as well as a water control.

**Additional file 3.** Phylogenetic relationships of the remaining viruses identified in this study. Sequences identified from the diseased group are marked with a red solid circle, whereas those from healthy controls are marked with a blue solid circle. For clarity, sequence names are not shown on the tree. The corresponding taxonomy/lineage and geographic information are provided on the right of the tree.

**Additional file 4.** Distribution of disease symptoms associated with each pathogen. For each pathogen a bar graph shows the number of cases (x axis) associated with each symptoms (y axis).

**Additional file 5.** Primer and probe sequence information.

## Data Availability

All data generated or analyzed during this study are included in the supplementary information files.

## Abbreviations

ASFV: African swine fever virus;
PRRSV: Porcine reproductive and respiratory syndrome virus
CSFV: Classical swine fever virus
PEDV: Porcine epidemic diarrhea virus
PRV: Pseudorabies virus
PPV: Porcine parvovirus
PCV2: Porcine circovirus 2
swine IAV: Swine influenza A virus
SPF: Specific pathogen free
HEV: Hepatitis E virus
RVA: Rotavirus A
JEV: Japanese encephalitis virus
GMS: Grocott’s methenamine silver
PHEV: Porcine hemagglutinating encephalomyelitis virus
RPM: Reads per million
aLRT: Approximate likelihood ratio test
