# Supplementary figures and images for: A total infectome approach to understand the etiology of infectious disease in pigs
Source: Microbiome. 2022 May 10;10:73. doi: 10.1186/s40168-022-01265-4 (PMC9086151; doi:10.1186/s40168-022-01265-4)

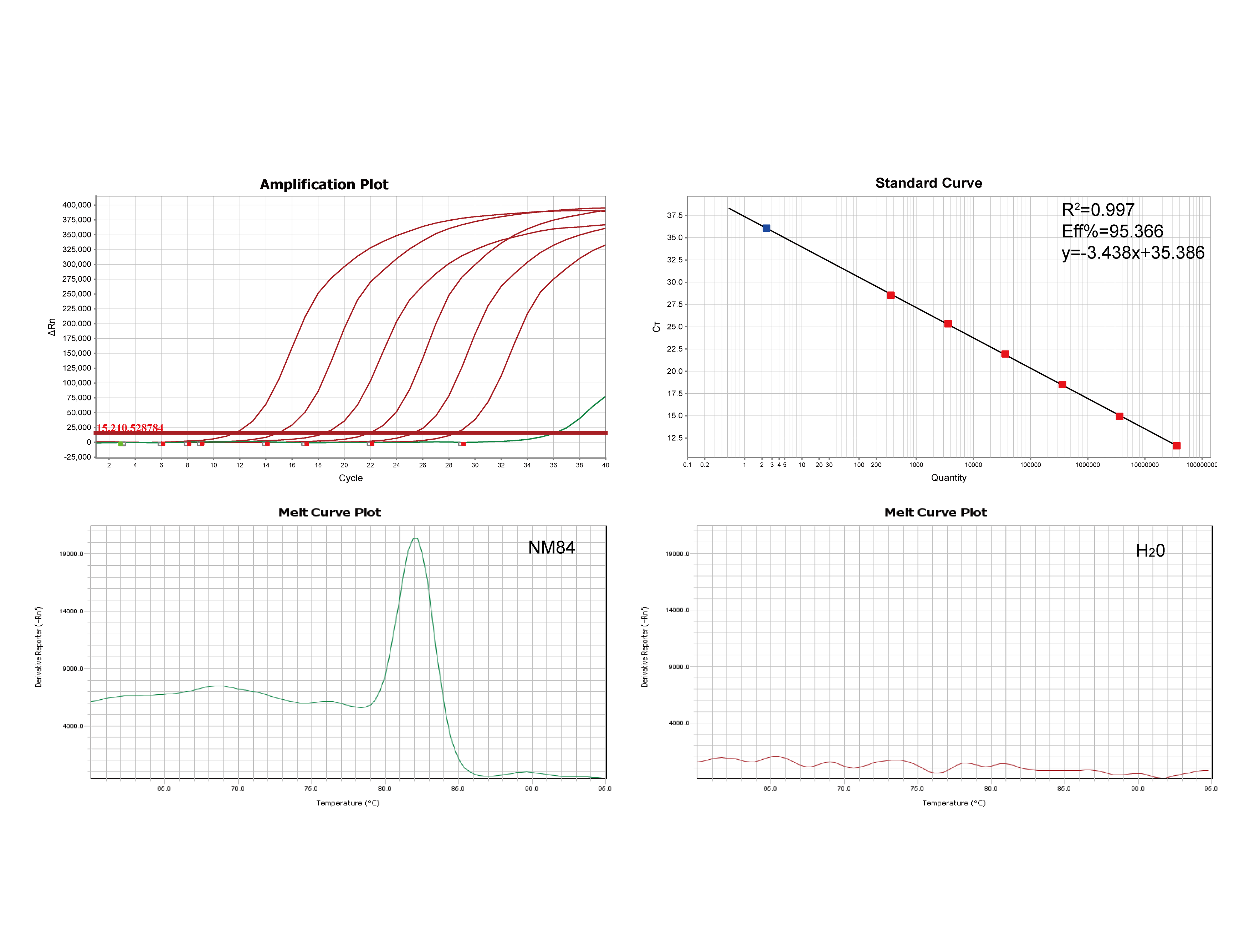

Supplement: Supplementary file 3 — Additional file 2. RT-qPCR result showing the presence of JEV in sample NM84. Top panel: amplification plot and standard curve based on absolute standards as well as in sample NM84 (green curve and blue square). Bottom panel: Melt curve plots for the sample as well as a water control. [file 40168_2022_1265_MOESM3_ESM.tif]

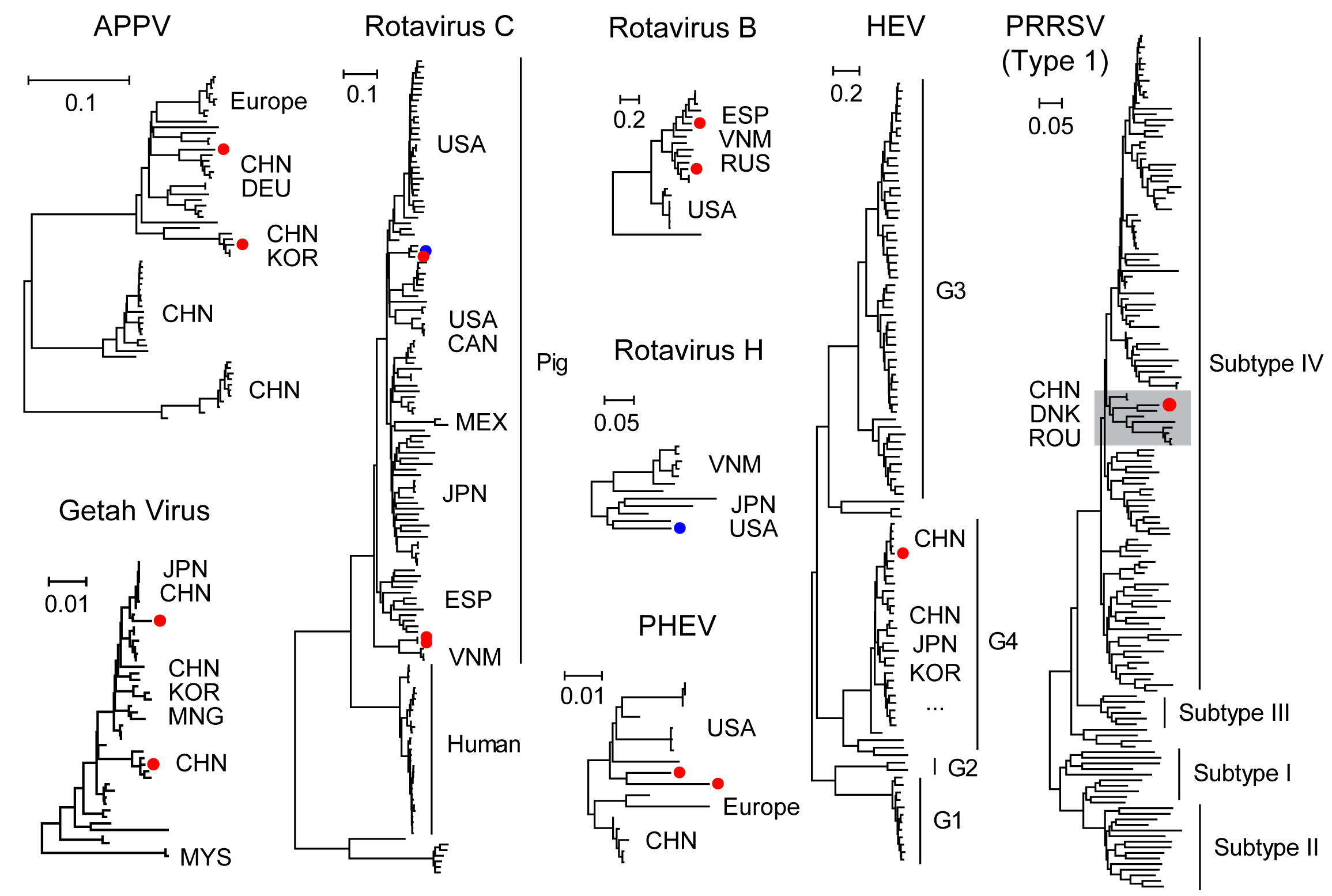

Supplement: Supplementary file 4 — Additional file 3. Phylogenetic relationships of the remaining viruses identified in this study. Sequences identified from the diseased group are marked with a red solid circle, whereas those from healthy controls are marked with a blue solid circle. For clarity, sequence names are not shown on the tree. The corresponding taxonomy/lineage and geographic information are provided on the right of the tree. [file 40168_2022_1265_MOESM4_ESM.tif]

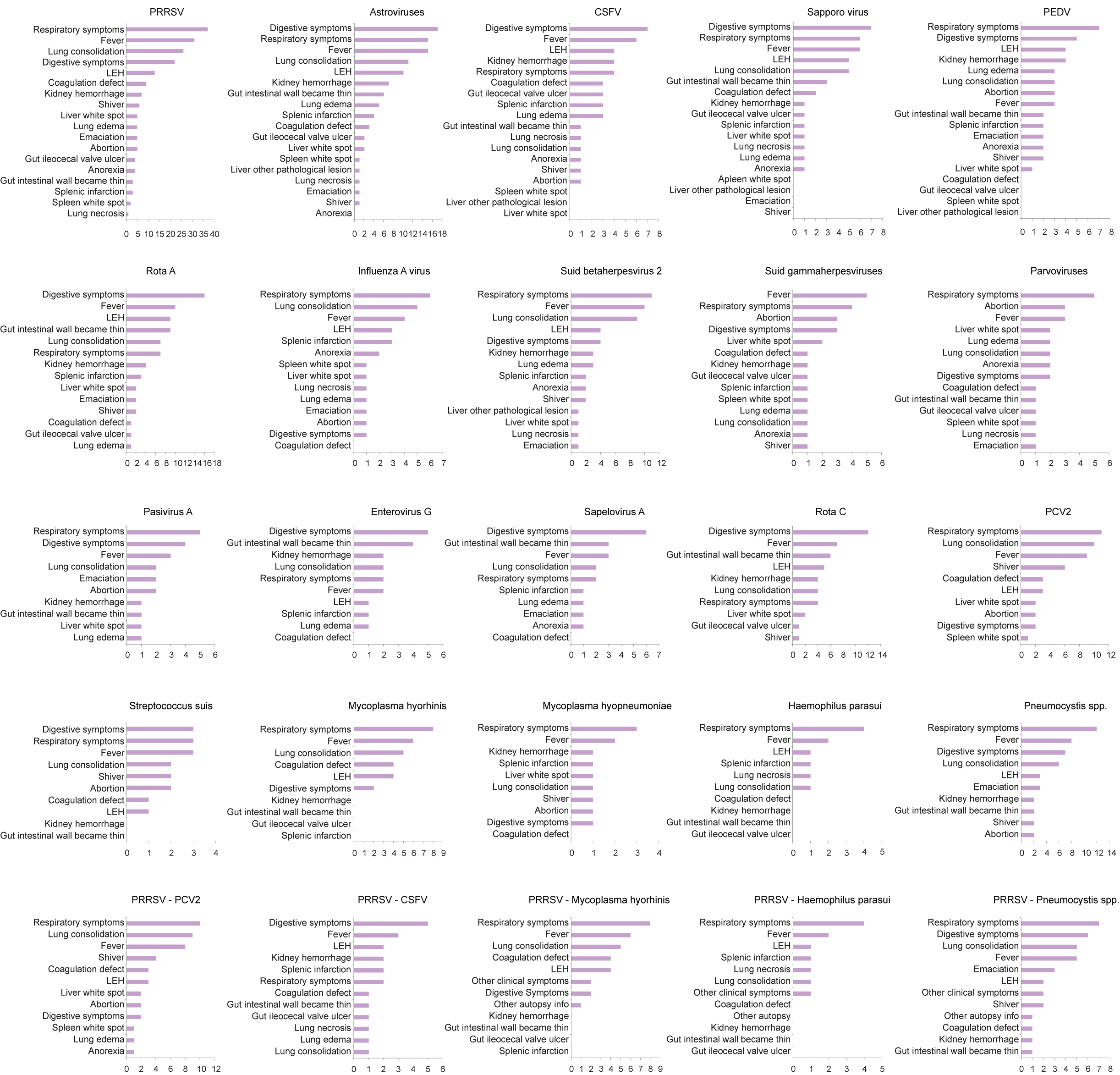

Supplement: Supplementary file 5 — Additional file 4. Distribution of disease symptoms associated with each pathogen. For each pathogen a bar graph shows the number of cases (x axis) associated with each symptoms (y axis). [file 40168_2022_1265_MOESM5_ESM.tif]
