# Supplementary material for: A total infectome approach to understand the etiology of infectious disease in pigs
Source: Microbiome. 2022 May 10;10:73. doi: 10.1186/s40168-022-01265-4 (PMC9086151; doi:10.1186/s40168-022-01265-4)
Supplement: Supplementary file 6 — Additional file 5. Primer and probe sequence information. [file 40168_2022_1265_MOESM6_ESM.docx]

Table S2. Primer/probe sequence information for RT-qPCR or qPCR assay.

| Primer/probe name | Sequence (5’-3’) | Target pathogen | Reference |
| --- | --- | --- | --- |
| *Pneumocystis*-forward | TAGCTGGTTTTCTGCGAAAT | *Pneumocystis* spp. | Weissenbacher-Lang et al., 2016 |
| *Pneumocystis*-reverse | TTCTGGGCTGTTTCCCTTTA |  |  |
| PRRSV1-UF | CAGATGCAGATTGTGTTGCCT | EU-type PRRSV | Chen et al., 2019 |
| PRRSV1-UR | ATGGAGACCTGCAGCACTTTC |  |  |
| PRRSV2-UF | TTGTGCTTGCTAGGCCGC | NA-type PRRSV |  |
| PRRSV2-UR | ACGACAAATGCGTGGTTATCA |  |  |
| PRRSV-probe | FAM-TCTGGCCCCTGCCCA-MGB |  |  |
| PRV gE-forward | TCGTGATGACGTGCGTCGTCG | PRV | Yoon et al., 2006 |
| PRV gE-reverse | CGCGGAACCAGTCGTCGAAGC |  |  |
| PRV gE-probe | FAM-CTACGAGGGGCCGTACGCG  AGCCTGGA-TAMRA |  |  |
| JEV-F | AGAGCGGGGAAAAAGGTCAT | JEV | Santhosh et al., 2007 |
| JEV-R | TTTCACGCTCTTTCTACAGT |  |  |
